# Clinical research on traditional Chinese medicine for Postherpetic neuralgia: an evidence map

**DOI:** 10.3389/fneur.2025.1633362

**Published:** 2025-10-08

**Authors:** Yongyuan Luo, Tingyu Yang, Zhongxi Hong, Chen Huang, Xiya Xiong, Xianyu Zhou, Xuemei An

**Affiliations:** ^1^Department of Nursing, Chengdu University of Traditional Chinese Medicine, Chengdu, China; ^2^Hospital of Chengdu University of Traditional Chinese Medicine, Chengdu, China; ^3^Deyang Hospital Affiliated to Chengdu University of Traditional Chinese Medicine, Deyang, China

**Keywords:** evidence map, PHN, Postherpetic neuralgia, traditional Chinese medicine, TCM

## Abstract

**Objective:**

This evidence mapping review evaluated clinical research on traditional Chinese medicine (TCM) for postherpetic neuralgia (PHN) to identify evidence distribution and gaps, thereby informing future studies.

**Methods:**

A systematic search was conducted in PubMed, Embase, Web of Science, the Cochrane Library, CBM, CNKI, and Wanfang databases for randomized controlled trials (RCTs) and systematic reviews (SRs)/meta-analysis (MAs) published up to December 2024. Data were extracted on publication trends, sample sizes, TCM pattern types, intervention types and duration, outcome indicators, and methodological quality. The evidences were analyzed and presented through a combination of text and graphical formats.

**Results:**

A total of 976 RCTs and 24 SRs/MAs were included. RCTs showed fluctuating growth, yet typically had small samples (51–100 cases). *Qi* stagnation and blood stasis was the most common TCM pattern. The main intervention was multi-therapy combination, with treatment duration primarily between 4 and 8 weeks. Outcome indicators mainly focused on total effective rate, pain intensity, and safety evaluation, while neglecting pain area, self-care ability, and neurotrophic factors. Furthermore, methodological quality assessment revealed suboptimal design rigor across both RCTs and SRs/MAs.

**Conclusion:**

TCM shows potential benefits for PHN but faces challenges in study design and outcome standardization. Future research should prioritize multicenter, large-scale RCTs with rigorous methodologies and harmonized outcome assessments. Meanwhile, enhancing the quality of SRs/MAs and integrating evidence-based frameworks will help bridge clinical practice with evidence-based TCM for PHN.

## 1 Introduction

Herpes zoster (HZ), known in traditional Chinese medicine (TCM) as “snake-like sore” or “snake-coiled fiery Dan,” is an acute self-limiting dermatological disorder resulting from the reactivation of the varicella-zoster virus (VZV) (1). It is characterized by vesicular eruptions distributed along affected dermatomes, often accompanied by acute pain (2). Postherpetic neuralgia (PHN), termed “lingering pain after snake-like sore” in modern TCM, arises from unresolved HZ and represents one of the most common complications of the disease (3). Although the precise temporal definition of PHN remains controversial, it is generally defined as pain persisting for more than 3 months after rash resolution (4, 5). The development of PHN is influenced by multiple factors, including female gender, advanced age, rash severity (widespread distribution, elevated local skin temperature), and prodromal pain (prolonged duration, intensified pain) (6, 7). PHN-related pain typically manifests as burning, cutting, or continuous/intermittent stabbing sensations (5), and is frequently accompanied by sensory abnormalities such as dysesthesia, hypoesthesia, and hyperalgesia within affected dermatomes (8). These symptoms occur in over 70% of PHN patients (9), may persist for months to years (10), and can severely impair health-related quality of life, including daily activities, social interaction, psychological well-being, and self-care capacity (4, 11).

The pathogenesis of PHN remains incompletely elucidated, with current evidence pointing predominantly to abnormal peripheral nerve conduction and central nervous system sensitization (12). Consequently, Western medical management of PHN emphasizes multimodal analgesia, often combining analgesics with neurotrophic agents. First-line pharmacological treatments include anticonvulsants (e.g., pregabalin and gabapentin), tricyclic antidepressants (e.g., amitriptyline), and topical lidocaine gel, whereas opioid analgesics and topical capsaicin are generally considered second- or third-line options (13). Non-pharmacological approaches involve minimally invasive treatments such as pulsed radiofrequency and nerve blocks, alongside psychological and physical adjunctive therapies (14).

Although pharmacological interventions provide rapid symptomatic relief for PHN patients, long-term use may carry risks of dependency and adverse effects such as dizziness, drowsiness, and edema (13, 15). Furthermore, monotherapy often yields suboptimal outcomes—first-line agents like gabapentin or pregabalin demonstrate response rates of only 35–40% as standalone treatments (16), frequently necessitating combination regimens to achieve adequate analgesia. However, such intensification increases the risk of cumulative adverse effects, particularly in elderly patients. Pharmacokinetic studies indicate an age-related decline in hepatic metabolism, resulting in a 10–40% reduction in drug clearance for most medications in older adults (17, 18), thereby elevating susceptibility to drug toxicity and underscoring the need for therapeutic monitoring in this population (19). Minimally invasive interventions, while offering benefits such as minimal trauma and few complications, face limitations due to high cost, uncertain long-term efficacy, and insufficient high-quality evidence regarding safety. A rigorous risk–benefit evaluation is therefore essential before implementation (20). Physical therapies can provide supplementary pain relief but are mechanistically constrained to a supportive, rather than curative, role. Similarly, psychological approaches may alleviate mental distress yet cannot substitute for primary treatments.

According to the collateral disease theory in TCM, the pathogenesis of PHN is broadly categorized into “obstruction-induced pain” and “deficiency-induced pain,” corresponding to excess (*shi*) and deficiency (*xu*) patterns, respectively (21). The excess pattern is characterized by the retention of dampness, heat, and toxins, leading to obstruction of *qi* and blood circulation and resulting in stasis within the collaterals (22). In contrast, the deficiency pattern arises from prolonged retention of pathogenic factors, which consume *qi* and blood, thereby depriving the *zang-fu* organs and meridians of nourishment (22). Consequently, TCM management of PHN follows the principle of “purging excess and tonifying deficiency,” with treatment strategies emphasizing pattern differentiation and focusing on resolving collateral stasis and promoting *qi* and blood circulation (23). For example, a meta-analysis by Kui et al. (24) showed that bloodletting puncture and cupping therapy can alleviate PHN-related pain by stimulating acupoints, unblocking meridians, and regulating *qi* and blood flow.

Rooted in a profound historical foundation and a well-developed theoretical system, TCM offers valuable approaches for managing dermatological diseases (25). Growing evidence supports the efficacy of TCM in PHN treatment, with advantages including diverse therapeutic modalities, few adverse effects, and wide clinical applicability (26). Nonetheless, the overall clinical evidence for various TCM interventions remains inconclusive, and a comprehensive synthesis of existing research in this field is still lacking.

Evidence mapping is a systematic approach for synthesizing and critically evaluating evidence within a specified research field (27). It employs visual analytics to transform complex information, such as current research status, challenges, and development trends, into structured graphical overviews, providing evidence users with a comprehensive perspective to enhance research effectiveness and practical impact (28). This study utilized evidence mapping to summarize high-level evidence on TCM interventions for PHN, including randomized controlled trials (RCTs) and systematic reviews/meta-analyses (SRs/MAs). Through the multidimensional visualization of evidence matrices, it aimed to assess the current state and limitations in the field. Thus, it serves both as a decision-support tool for healthcare administrators and a methodological reference for future standardization efforts in TCM.

## 2 Methods

This study developed an evidence map based on the methodological framework of Miake-Lye et al. (28), which outlines the core elements of systematic evidence mapping, including systematic search, evidence gap identification, visual presentation, stakeholder involvement, and methodological rigor. The process was further supplemented by following the PRISMA-ScR guidelines proposed by Tricco et al. (29), enhancing both its reporting quality and methodological soundness.

### 2.1 Literature inclusion and exclusion criteria

The inclusion criteria were: (1) study population: patients diagnosed with PHN based on both Western medical and TCM pattern differentiation criteria. No restrictions were applied regarding gender, age, ethnicity, disease duration, or comorbidities; (2) interventions: the experimental group received TCM therapies, integrated TCM and Western treatment, or TCM-based nursing care. The control group received conventional Western medicine (CWM), physical therapy, placebo or standard care; (3) study designs: published RCTs and SRs/MAs investigating TCM interventions for PHN; (4) languages: Chinese and English.

The exclusion criteria were: (1) full text unavailable; (2) duplicate publications; (3) studies with incomplete or preliminary data (e.g., study protocols or conference abstracts); (4) non-compliant study designs, such as non-randomized trials, unmatched subject groups, or inappropriate control groups; (5) animal experiments, case reports, experiential summaries, reviews, or unrelated article types.

### 2.2 Search strategy

A systematic search was conducted in PubMed, Embase, Web of Science, the Cochrane Library, CBM, CNKI, and Wanfang, covering publications up to December 2024. The search strategy combined MeSH terms and free-text words, with keywords such as “Neuralgia, Postherpetic,” “Postherpetic Neuralgia,” “Post-herpetic Neuralgia,” “PHN,” “Medicine, Chinese Traditional,” “Traditional Chinese Medicine,” “TCM,” “Acupuncture,” “Moxibustion,” “Cupping,” etc. Adjusted the search strategy according to the characteristics of each database. Additionally, the reference lists of relevant studies were retrieved manually (Supplementary Table S1).

### 2.3 Literature screening and data extraction

Two researchers independently performed literature screening, data extraction, and cross-verified findings based on pre-defined inclusion and exclusion criteria. Discrepancies were resolved through discussion or third-party consultation. The process involved: (1) using NoteExpress software for reference management and duplicate removal; (2) excluding obviously irrelevant literature after reading titles and abstracts; (3) critically reviewing the full texts of eligible studies and assessing their methodological quality; (4) documenting detailed reasons for exclusion; (5) extracting data using a pre-defined form: first author, publication year, sample size, TCM pattern type, interventions and duration, outcome indicators, and efficacy.

### 2.4 Methodological quality assessment

Two researchers independently assessed the methodological quality of the included studies using appropriate tools based on study design. Disagreements were resolved through discussion or by consulting a third reviewer.

#### 2.4.1 Assessment of RCTs

The Cohrane Risk of Bias Tool (30) was applied to evaluate the following aspects: (1) random sequence generation; (2) allocation concealment; (3) blinding of participants and personnel; (4) blinding of outcome assessment; (5) completeness of outcome data; (6) selective reporting; (7) other potential biases. Each item was rated as “low risk,” “high risk,” or “unclear.”

#### 2.4.2 Assessment of SRs/MAs

The AMSTAR-2 Scale (31), which consists of 16 items, was used for appraisal. Seven of these items (2, 4, 7, 9, 11, 13, and 15) were considered critical to the rigor and validity of the SRs. Each item was scored as “yes,” “no,” or “partial yes.” A study was classified as having adequate reporting if ≥ 70% of items received a “yes” or “partial yes.” Based on the ratings, the overall quality was categorized into four levels: “high quality” (no critical flaws, and ≤ 1 non-critical item with “no”); “moderate quality” (no critical flaws, but > 1 non-critical item with “no”); “low quality” (one critical flaws, with ≤ 1 non-critical item rated “no”); “critically low quality” (> 1 critical flaws, regardless of non-critical items).

### 2.5 Statistical analysis

Data analysis combined graphical representations and textual descriptions. Specifically, the literature screening process was illustrated with a flowchart, annual publication trends were shown with a line chart, intervention duration was displayed as a bar chart, and quality assessment results were presented in a strip chart. Category distributions were visualized with pie charts or three-line tables, and evidence distributions via bubble charts. Furthermore, the bubble chart categories were derived inductively from the predominant interventions and outcomes in the included studies.

The clinical efficacy reported in the included SRs/MAs was classified as follows (32): “beneficial” (when results and conclusions consistently indicated a clear beneficial effect of the intervention, supported by studies with low risk of bias); “probably beneficial” (when a positive effect was reported but conclusions did not explicitly confirm benefit, or results were non-significant yet implied potential benefits); “harmful” (when both results and conclusions indicated adverse effects); “no differential effect” (when no significant differences were found between intervention and control groups); and “inconclusive” (when evidence was insufficient to determine either a definite or probable effect).

## 3 Results

### 3.1 Literature screening process

**Figure 1:**
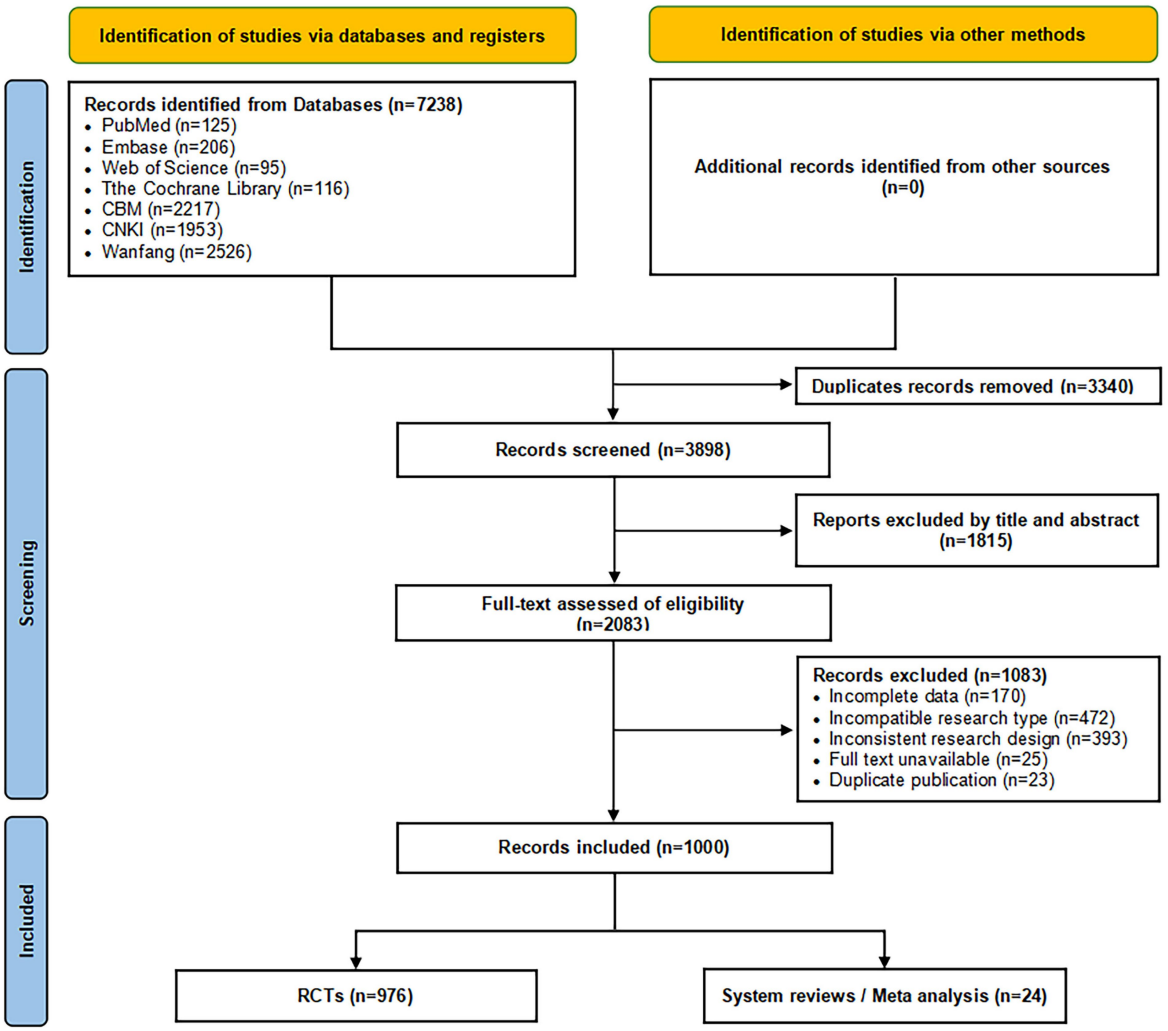
Literature screening process and results.

Based on pre-defined inclusion and exclusion criteria, a comprehensive search of seven Chinese and English databases identified 7,238 articles. After duplicate removal, initial screening, and re-screening, a final set of 1,000 publications were ultimately included for analysis, consisting of 976 RCTs and 24 SRs/MAs. The literature screening process and results are illustrated in Figure 1.

### 3.2 Publication trends

**Figure 2:**
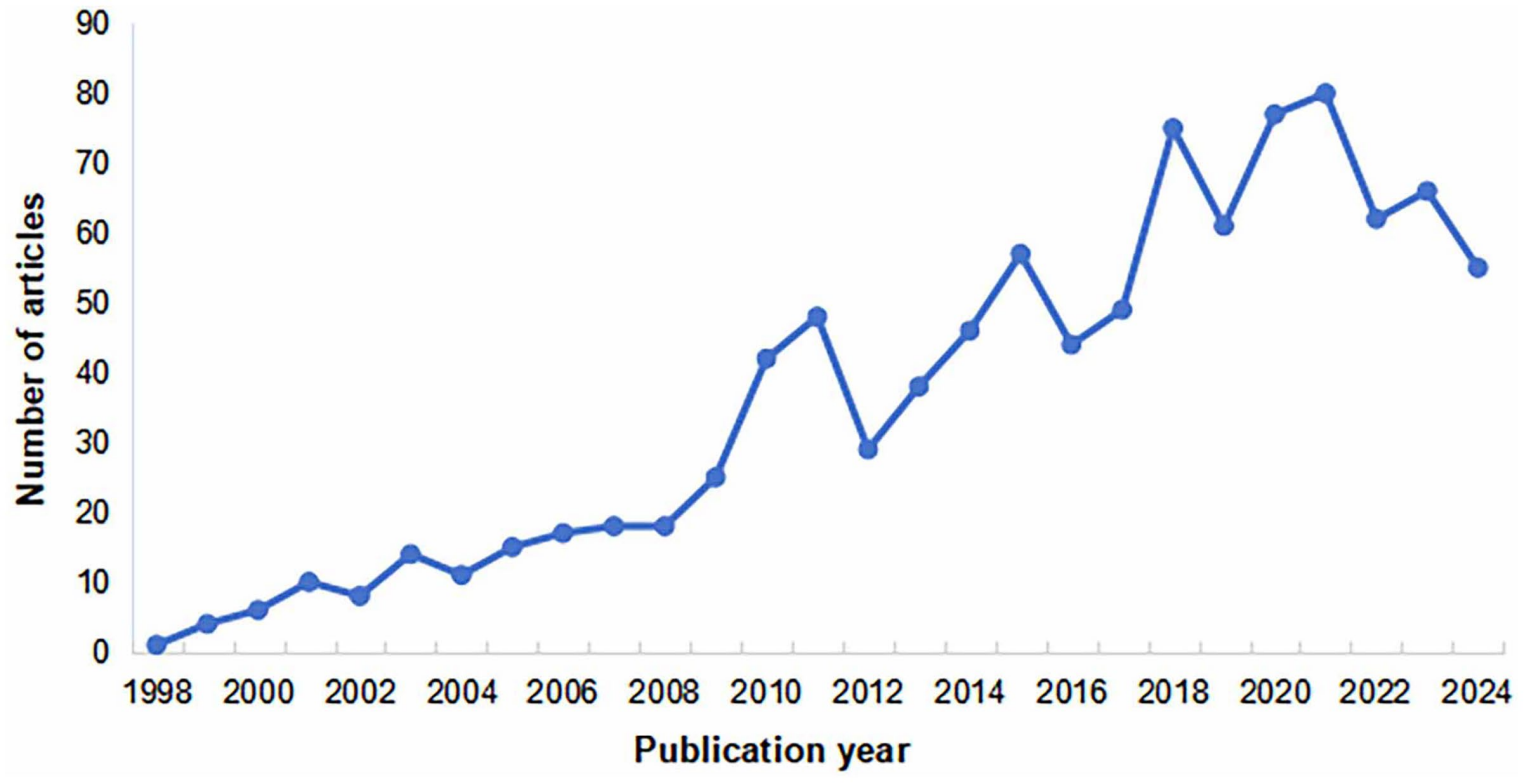
Annual publication trends of RCTs on TCM for PHN.

The initial phase (1998–2008) was characterized by a limited yet steadily rising number of publications, reflecting the nascent stage of the field and a gradually growing academic attention. This was followed by a period of rapid expansion (2009–2021), which peaked in 2021, indicating increasing recognition of TCM’s benefits for treating PHN alongside its broader application in pain management. Although a slight decline occurred from 2022 to 2024, the overall trend maintains sustained—albeit fluctuating—growth, suggesting that ongoing advances in TCM and improved clinical research methodologies will foster more in-depth and extensive development in this field. As shown in Figure 2.

### 3.3 Sample sizes

Analysis of the sample sizes in the included RCTs revealed a range from 17 to 394 cases. The majority of studies (*n =* 714) enrolled 51–100 cases, followed by 101–200 (*n =* 133) and ≤ 50 cases (*n =* 114), while relatively few exceeded 200. The distribution is summarized in Table 1.

**Table 1:** Sample sizes of RCTs on TCM for PHN.

Study sample sizes (*n*)	Number of articles (*n*)	Proportion (%)
≤ 50	114	11.68
51 ~ 100	714	73.15
101 ~ 200	133	13.63
201 ~ 300	13	1.33
> 300	2	0.21

### 3.4 TCM pattern classifications of the subjects

TCM patterns represent the generalization and synthesis of a disease’s progression at specific pathological stages (33). These patterns manifest through clinical symptoms, tongue and pulse characteristics, and constitutional presentations, collectively revealing the etiology, lesion location, pathogenic nature, and the dynamic balance between pathogenic factors and vital qi (34). As the core of TCM diagnosis, pattern differentiation integrates four diagnostic methods (inspection, auscultation, inquiry, and palpation) to discern disease nature and pattern types (35), forming a essential basis for TCM treatment (36).

**Figure 3:**
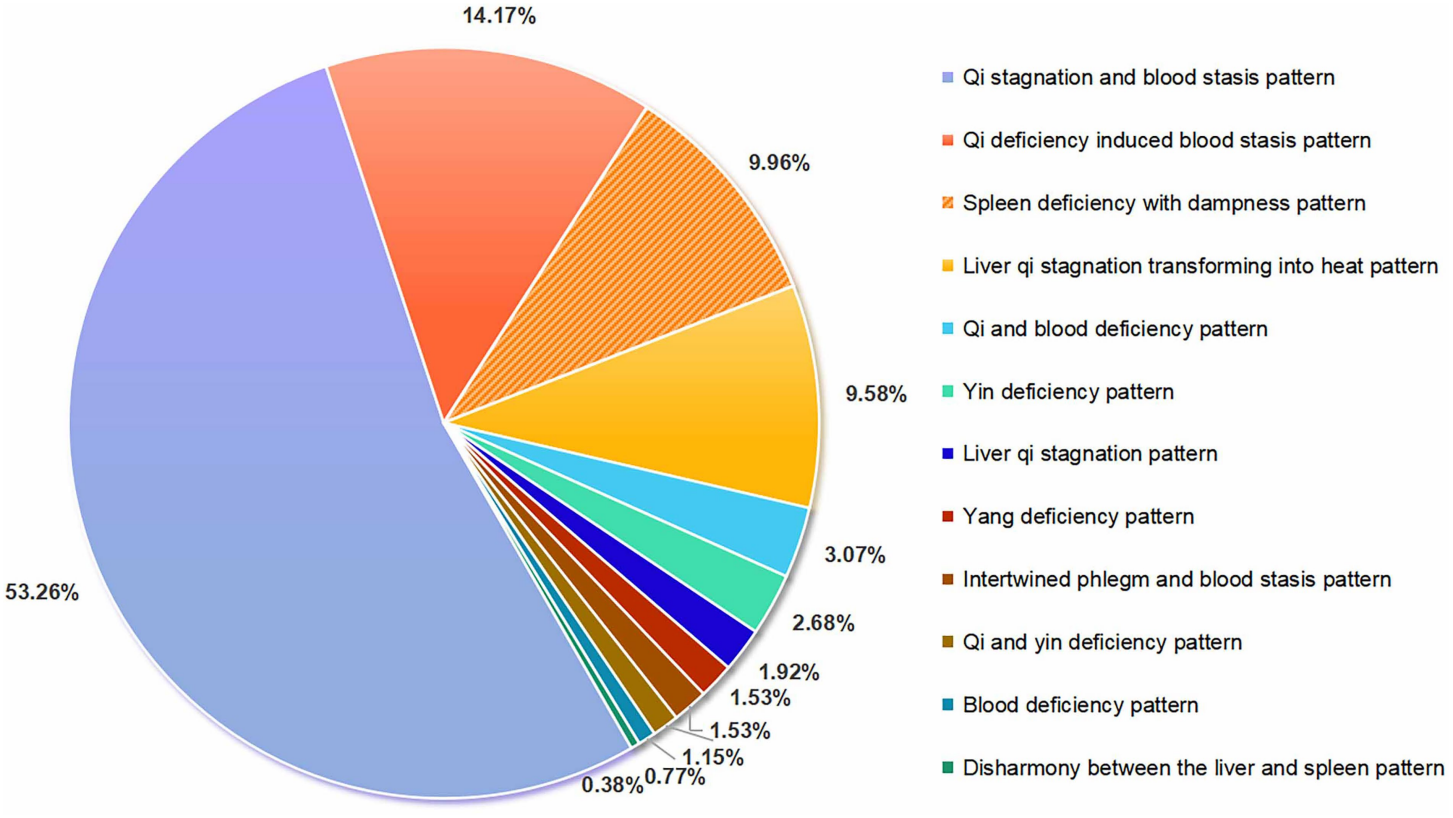
TCM pattern classifications of the subjects.

There is no universally accepted standard for the TCM pattern differentiation of PHN, though many experts associate its pathogenesis with dampness, heat, fire, and toxic pathogens (37). This study classified TCM patterns according to the “Guideline for TCM Diagnosis and Treatment of Snake-Like Sores (2014 Revision)” (38) and “Diagnostic and Efficacy Criteria for TCM Diseases and Syndromes” (39). Among the 976 included RCTs, 199 reported specific TCM pattern types. The predominant diagnostic pattern was “*qi* stagnation and blood stasis” (characterized by fixed stabbing pain and inhibited blood flow; WHO-IST code 1126; 53.26%), followed by “*qi* deficiency induced blood stasis” (fatigue with fixed severe pain; WHO-IST code 1134; 14.17%), “spleen deficiency with dampness” (digestive symptoms with heaviness/edema; WHO-IST code 1205; 9.96%), and “liver *qi* stagnation transforming into heat” (burning subcostal pain with dry mouth; WHO-IST code 1244; 9.58%). Refer to Figure 3.

All TCM pattern terms and definitions align with the “WHO International Standard Terminologies on Traditional Chinese Medicine(2022)”(WHO-IST 2022) (40), with full descriptions available in Supplementary Table S2.

### 3.5 Intervention types and duration

#### 3.5.1 Intervention types

**Figure 4:**
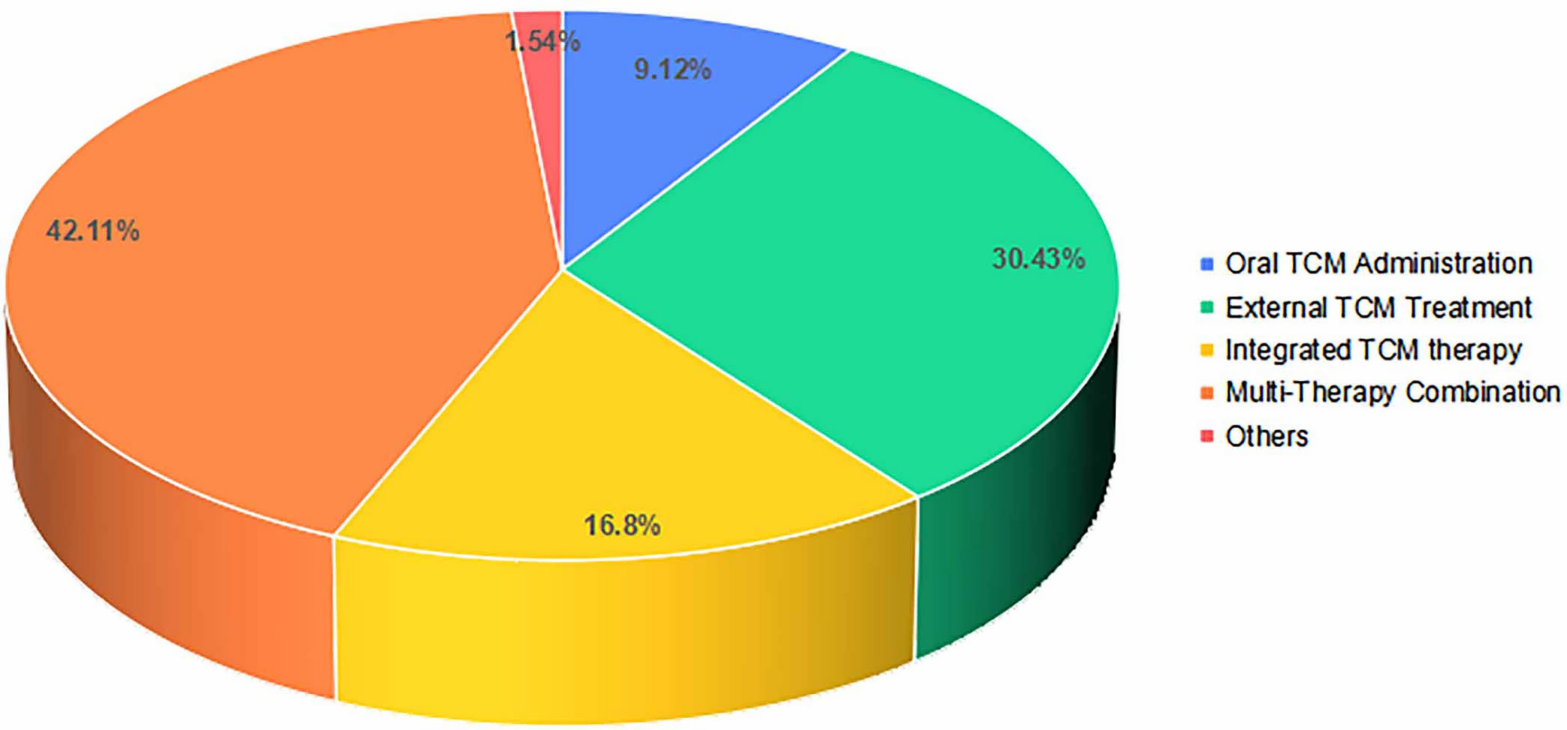
The distribution of intervention types of RCTs on TCM for PHN.

The included RCTs featured five therapeutic modalities: oral TCM administration, external TCM treatment, integrated TCM therapy, multi-therapy combination and others. Multi-therapy combination, defined as one or more TCM interventions combined with CWM, interventional therapy, or physical therapy, represented the largest category (42.11%, *n =* 411), reflecting a clinical preference for comprehensive strategies that may enhance efficacy and reduce adverse effects by targeting the multifactorial pathogenesis of PHN (41). External TCM treatment, which encompasses all body-surface therapies such as external application, fumigation, acupuncture, bloodletting, moxibustion, and cupping, constituted the second most common modality (30.43%, *n =* 297). Integrated TCM therapy, referring to the concurrent or sequential use of oral and external TCM treatments (e.g., oral herbs plus acupuncture or cupping), accounted for 16.80% (*n =* 164), ranking third. As illustrated in Figure 4.

Among the 364 RCTs investigating oral TCM administration, the primary therapeutic strategies involved “circulating blood and transforming stasis,” “transforming stasis and unblocking collaterals,” “soothing the liver and regulating *qi*,” “clearing heat and removing toxins,” as well as “calming the mind with heavy medicinal minerals.” Research on TCM decoctions comprised 44 self-formulated prescriptions and 45 classic formulas, with “*Taohong Siwu* Decoction,” “*Xuefu Zhuyu* Decoction,” “*Buyang Huanwu* Decoction,” “*Chaihu Shugan* Powder,” “*Longdan Xiegan* Decoction,” “Paeoniae and Glycyrrhizae Decoction,” and “*Fuyuan Huoxue* Decoction” being the most frequently used due to their established clinical efficacy. Additionally, research on proprietary Chinese medicines covered 18 formulations, including “*Xuefu Zhuyu* Capsule,” “*Liushen* Pill,” “*Liuwei Dihuang* Pill,” and “*Fufang Danshen* Tablet,” among others, which also demonstrated notable clinical benefits. For detailed ingredients and therapeutic effects (see Supplementary Table S3).

Among the 297 studies on external TCM treatment, the most frequently reported interventions (≥ 5 studies each) were: acupuncture, acupuncture + bloodletting, bloodletting, acupuncture + acupoint injection, external application + acupuncture, acupuncture + cupping, moxibustion, bloodletting + moxibustion, external application, and acupoint injection. Of the 164 studies on integrated TCM therapies, the predominant combination (with frequency ≥ 5) were: oral TCM administration + acupuncture, oral TCM administration + bloodletting, oral TCM administration + external application, oral TCM administration + moxibustion, oral TCM administration + acupuncture + cupping. Among the 411 studies on multi-therapy combination, the most common interventions (≥ 5 occurrences) included: external application + Western medicine, oral TCM administration + Western medicine, integrated TCM therapy + Western medicine, external application + physical therapy, external application + interventional therapy, external application + Western medicine + interventional therapy, oral TCM administration + interventional therapy, oral TCM administration + physical therapy, external application + nursing, external application + Western medicine + physical therapy, external application + physical therapy + nursing, integrated TCM therapy + Western medicine + physical therapy. A complete frequency summary is provided in Table 2.

**Table 2:** Frequency analysis of different interventions.

Classification	Interventions	Frequency
External TCM Treatment	Acu.	143
	Acu. + Blt.	35
	Blt.	25
	Acu. + Acu. Inj.	16
	Ext. Appl. + Acu.	9
	Acu. + Cup.	8
	Mox.	7
	Blt. + Mox.	7
	Ext. Appl.	5
	Acu. Inj.	5
Integrated TCM Therapy	Oral TCM + Acu.	69
	Oral TCM + Blt.	33
	Oral TCM + Ext. Appl.	17
	Oral TCM + Mox.	7
	Oral TCM + Acu. + Cup.	7
Multi-Therapy Combination	Ext. TCM + WMT.	157
	Oral TCM + WMT.	85
	Integ. TCM + WMT.	38
	Ext. TCM + Phys.	37
	Ext. TCM + Interv.	15
	Ext. TCM + WMT. + Interv.	12
	Oral TCM + Interv.	11
	Oral TCM + Phys.	10
	Ext. TCM + Nursing	7
	Ext. TCM + WMT. + Phys.	7
	Ext. TCM + Phys. + Nursing	7
	Integ. TCM + WMT. + Phys.	5

Acu., Acupuncture; Blt., Bloodletting; Acu. Inj., Acupuncture Injection; Ext. Appl., External Application; Cup., Cupping; Mox., Moxibustion; Oral TCM, Oral TCM Administration; Ext. TCM Treat., External TCM Treatment; Integ. TCM, Integrated TCM Therapy; WMT, Western Medicine Treatment; Phys., Physical Therapy; Interv., Interventional Therapy.

#### 3.5.2 Intervention duration

**Figure 5:**
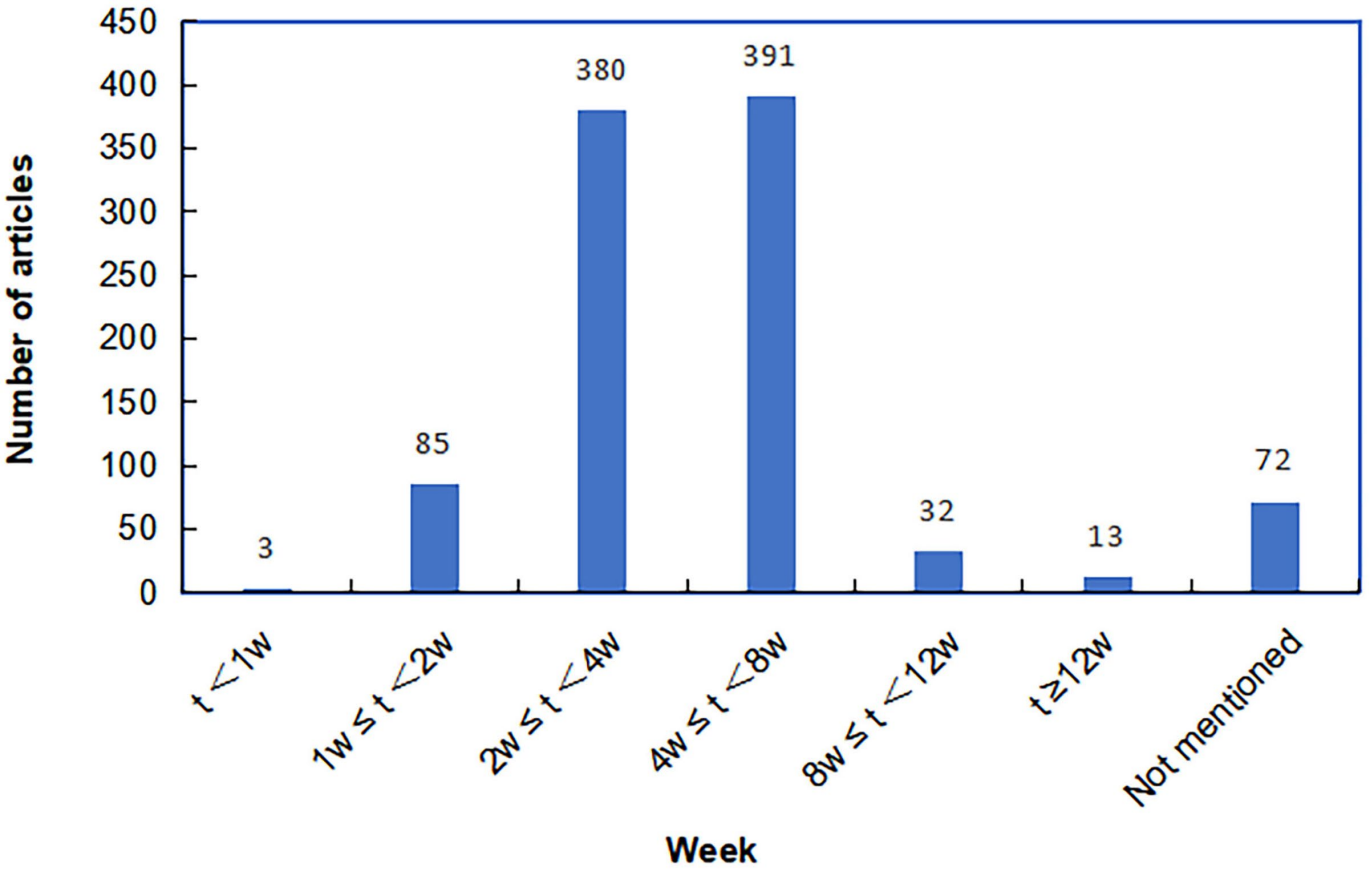
The distribution of intervention duration of RCTs on TCM for PHN.

Analysis of 976 RCTs revealed intervention duration ranging from 5 days to 6 months. Most studies implemented treatment courses of 4–8 weeks (*n =* 391) or 2–4 weeks (*n =* 380), followed by 1–2 weeks (*n =* 85) and 8–12 weeks (*n =* 32). Interventions lasting less than one week or beyond 12 weeks were infrequent, and 72 studies did not report specific duration (Figure 5).

The relatively short-term interventions (≤ 8 weeks) contrasts with the chronic nature of PHN, which often persists for months or longer (42, 43). Given that approximately 50% of PHN patients continue to experience moderate-to-severe pain even 9 months after rash onset (4), long-term pain management remains a critical challenge (44). However, few studies included follow-up observations, reflecting a general neglect of long-term prognosis in current research. The scarcity of longer-duration interventions and systematic follow-up impedes the evaluation of treatment sustainability. It is therefore recommended that future clinical trials, especially on TCM interventions, should align intervention length with the chronicity of PHN (e.g., at least 12 weeks) and include extended follow-up periods (ideally 12–24 weeks or more) to adequately assess lasting efficacy and long-term safety.

### 3.6 Evaluation of therapeutic efficacy

#### 3.6.1 Outcome indicators in RCTs

Therapeutic evaluation of TCM for PHN primarily involved the following 12 categories of indicators:

(1) clinical efficacy: total effective rate; cured cases; treatment sessions. (2) symptoms and signs: pain symptoms [pain area, pain episodes (mean daily frequency, mean duration per episode, mean time to pain relief, mean time to pain disappearance, mean interval between episodes, mean analgesic duration, analgesic usage/dosage), pain intensity (pain scores), pain threshold]; other clinical symptoms (e.g., numbness, burning sensation, itching, tactile hypersensitivity). (3) TCM syndrome patterns: TCM syndrome/symptom scores. (4) sleep quality: sleep quality scores; sleep efficiency; pain-related sleep interference; awakening frequency/duration; continuous/total sleep time. (5) emotional status: anxiety and depression scores. (6) quality of life: quality of life scores. (7) self-care ability: daily living/work ability scores. (8) laboratory parameters: inflammatory markers [interleukins (IL-2, IL-4, IL-6, IL-8, IL-10, IL-18, IL-1β), c-reactive protein (CRP), tumor necrosis factor-*α* (TNF-α), prostaglandin E2 (PGE2), cyclooxygenase-2 (COX-2), interferon-*γ* (IFN-γ), monocyte chemoattractant protein-1 (MCP-1), procalcitonin (PCT), CXC chemokine ligand 10 (CXCL10)]; immune markers [immunoglobulins (IgM, IgA, IgE, IgG), complement C3, complement C4, T lymphocyte subsets (CD3+, CD4+, CD8+, CD4+/CD8+, Th1/Th2, Th17, Treg)]; neurotrophic factors [brain-derived neurotrophic factor (BDNF), central nervous system-specific protein (S100*β*), neuron specific enolase (NSE), nerve growth factor (NGF)]; neurotransmitters and related peptides [serum substance P (SP), neuropeptide Y (NPY), β-endorphin (β-EP), 5-hydroxytryptamine (5-HT), neurokinin-1receptor (NK-1), calcitonin gene-related peptide (CGRP), transient receptor potential vanilloid subfamily 1 (TRPV1)]; kinases [p38 mitogen-activated protein kinase (MAPK), extracellular signal-regulated kinase (ERK), c-Jun N-terminal kinase (JNK)] (9) safety evaluation: adverse effects; tolerance; blood/urine/stool routine; liver/kidney function; electrocardiogram; electroencephalogram; rescue medication usage (10) economic indicators: mean length of hospital stay; treatment costs (11) long-term prognosis: recurrence rate; prognostic indices; follow-up symptom improvement (12) others: treatment compliance; satisfaction evaluation.

Among the assessment tools, the Visual Analog Scale (VAS) was predominantly employed to evaluate pain intensity (87.16%), while the Pittsburgh Sleep Quality Index (PSQI) provided a multidimensional assessment of sleep quality (55.73%). Anxiety and depression were frequently measured using the Self-rating Anxiety Scale (SAS) (48.78%), Self-rating Depression Scale (SDS) (52.44%), Hamilton Anxiety Rating Scale (HAMA) (46.34%), and Hamilton Depression Rating Scale (HAMD) (45.12%). Additionally, quality of life was commonly evaluated with the Dermatology Life Quality Index (DLQI, 41.28%) and the Short Form-36 Health Survey (SF-36, 35.78%).

**Figure 6:**
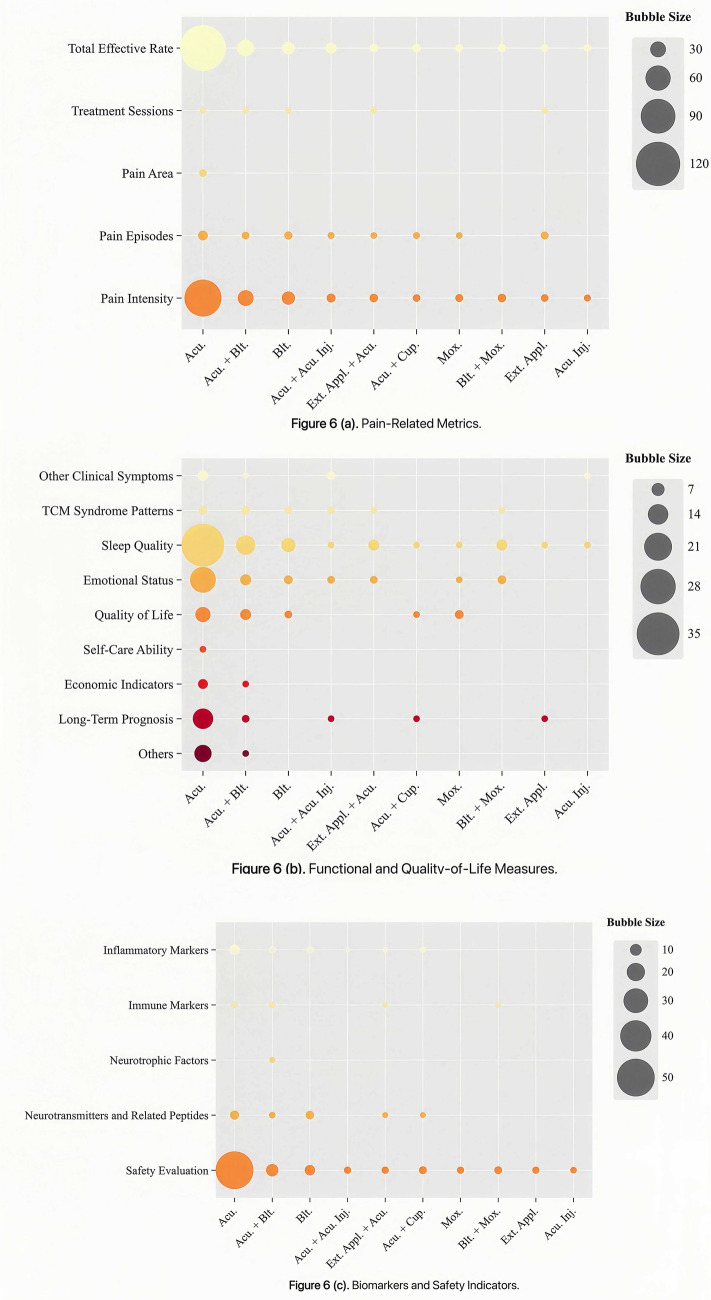
Evidence distribution of RCTs on external TCM treatment for PHN.

**Figure 7:**
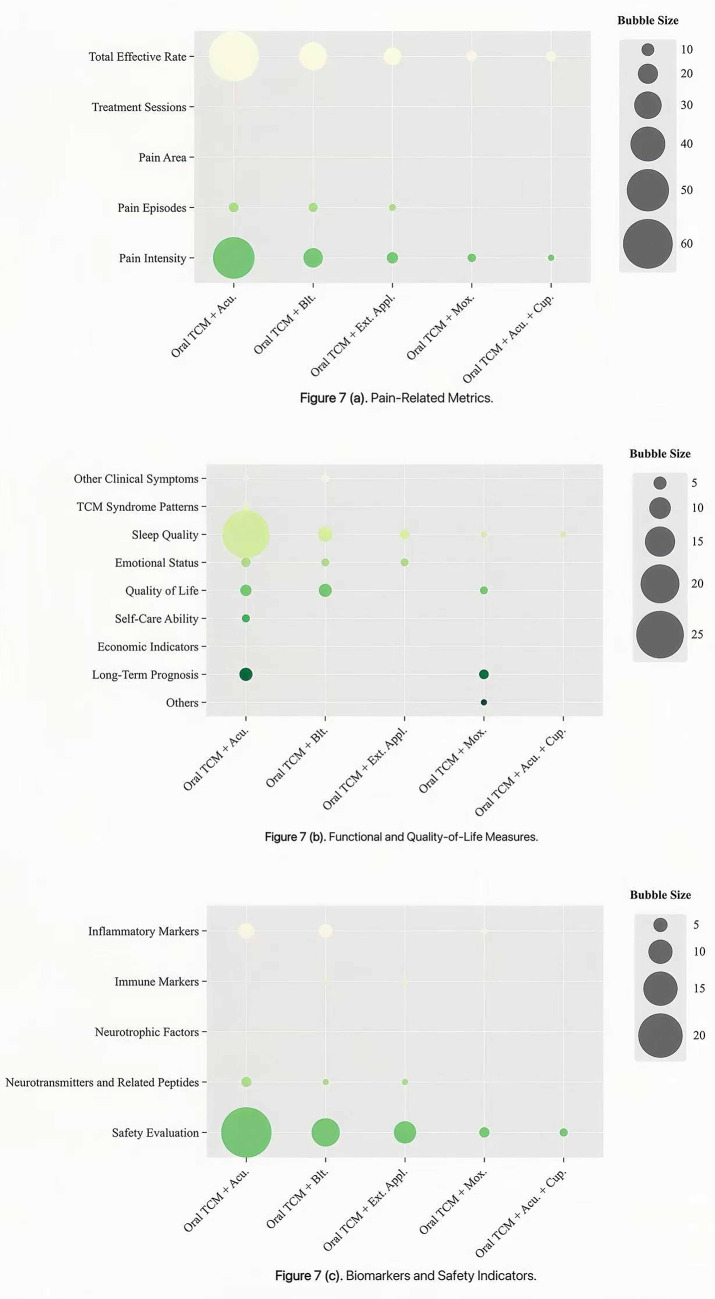
Evidence distribution of RCTs on integrated TCM therapy for PHN.

**Figure 8:**
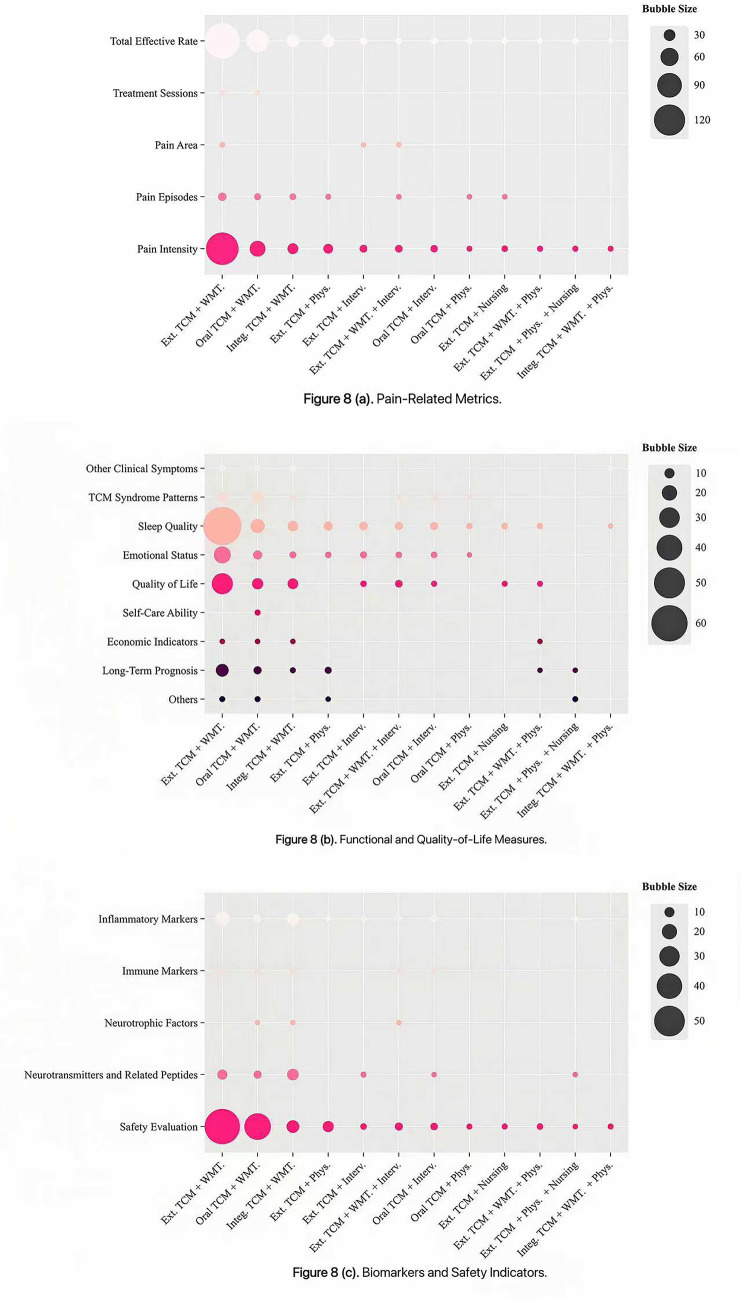
Evidence distribution of RCTs on multi-therapy combination for PHN.

Bubble chart analysis showed distinct patterns in outcome reporting across intervention types. For clarity, outcomes were categorized into three thematic groups: pain-related metrics, functional and quality-of-life measures, and biomarkers and safety indicators. The x-axis denotes intervention types, the y-axis represents outcome indicators, and bubble size corresponds to the number of included studies. Studies on external TCM treatments most frequently evaluated the total effective rate, pain episodes, pain intensity, sleep quality, emotional status and safety evaluation, whereas pain area, self-care ability, and neurotrophic factors were less commonly assessed (Figure 6). RCTs investigating integrated TCM therapies emphasized the total effective rate, pain intensity and safety evaluation, but showed notable gaps in reporting treatment sessions, pain area, TCM syndrome patterns, self-care ability, neurotrophic factors and economic indicators (Figure 7). Among multi-therapy combination studies, frequently reported outcomes included the total effective rate, pain intensity, sleep quality, emotional status, quality of life and safety evaluation, with self-care ability being the least measured outcome (Figure 8).

#### 3.6.2 Conclusions of the SRs/MAs

**Figure 9:**
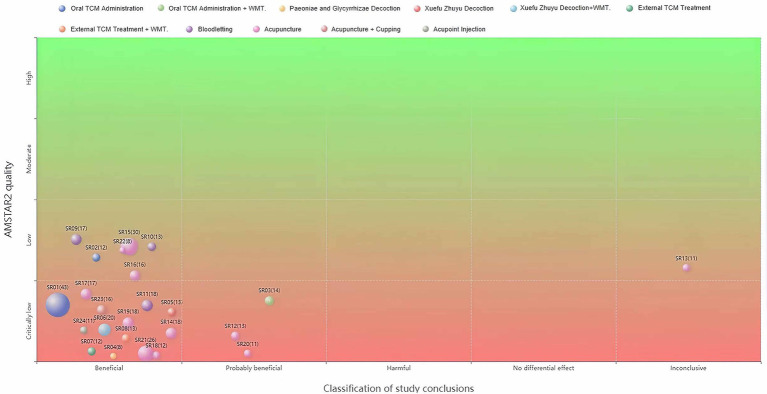
Evidence distribution for SRs/MAs. The x-axis indicates clinical efficacy categories, the y-axis shows methodological quality ratings, bubble size corresponds to the number of studies, and bubble color represents intervention types.

This study included 24 SRs/MAs. Control groups received CWM, physical therapy, placebo, or standard care, while experimental groups were treated with TCM therapy, integrated TCM and Western medicine, or TCM-specific nursing. Interventions among these studies comprised acupuncture (*n =* 11), bloodletting (*n =* 3), oral TCM administration (*n =* 2), acupoint injection (*n =* 1), acupuncture + cupping (*n =* 1), and oral TCM administration + Western medicine (*n =* 1). Additional interventions included *Paeoniae and Glycyrrhizae* decoction (*n =* 1), *Xuefu Zhuyu* decoction (*n =* 1), *Xuefu Zhuyu* decoction + Western medicine (*n =* 1), external application (*n =* 1), and external application + Western medicine (*n =* 1). Regarding clinical efficacy, 20 studies were rated as “beneficial,” 3 as “probably beneficial,” and 1 as “inconclusive.” These results are visualized in Figure 9.

Quantitative synthesis of outcomes classified as “beneficial” robustly demonstrates that TCM interventions enhance clinical efficacy, alleviate pain, and maintain a favorable safety profile in PHN management compared to CWM. The oral TCM administration, particularly compound formulations, significantly increased the total effective rate (OR = 5.02, 95% CI [4.00, 6.30]) and reduced pain intensity (SMD = −1.98, [−2.33, −1.63]). Notable efficacy was also observed with specific decoctions, such as *Paeoniae and Glycyrrhizae* decoction (OR = 9.08, [2.74, 30.04]) and *Xuefu Zhuyu* decoction (RR = 1.25, [1.18, 1.32]). External TCM treatments, including bloodletting (OR = 4.36, [3.05, 6.23]; WMD = −0.90, [−1.01, −0.78]) and various acupuncture techniques (OR range: 4.56–6.05; SMD/WMD range: −1.73 to −2.66), consistently outperformed CWM. Network meta-analysis further indicated that combined therapies (e.g., acupuncture with medication and moxibustion) may represent the most effective strategy. Importantly, these clinical benefits were accompanied by a favorable safety profile, with significantly fewer adverse events across interventions such as oral TCM administration (OR = 0.50, [0.37, 0.68]) and acupuncture (RR = 0.04, [0.01, 0.32]).

### 3.7 Methodological quality

#### 3.7.1 Quality assessment of RCTs

**Figure 10:**
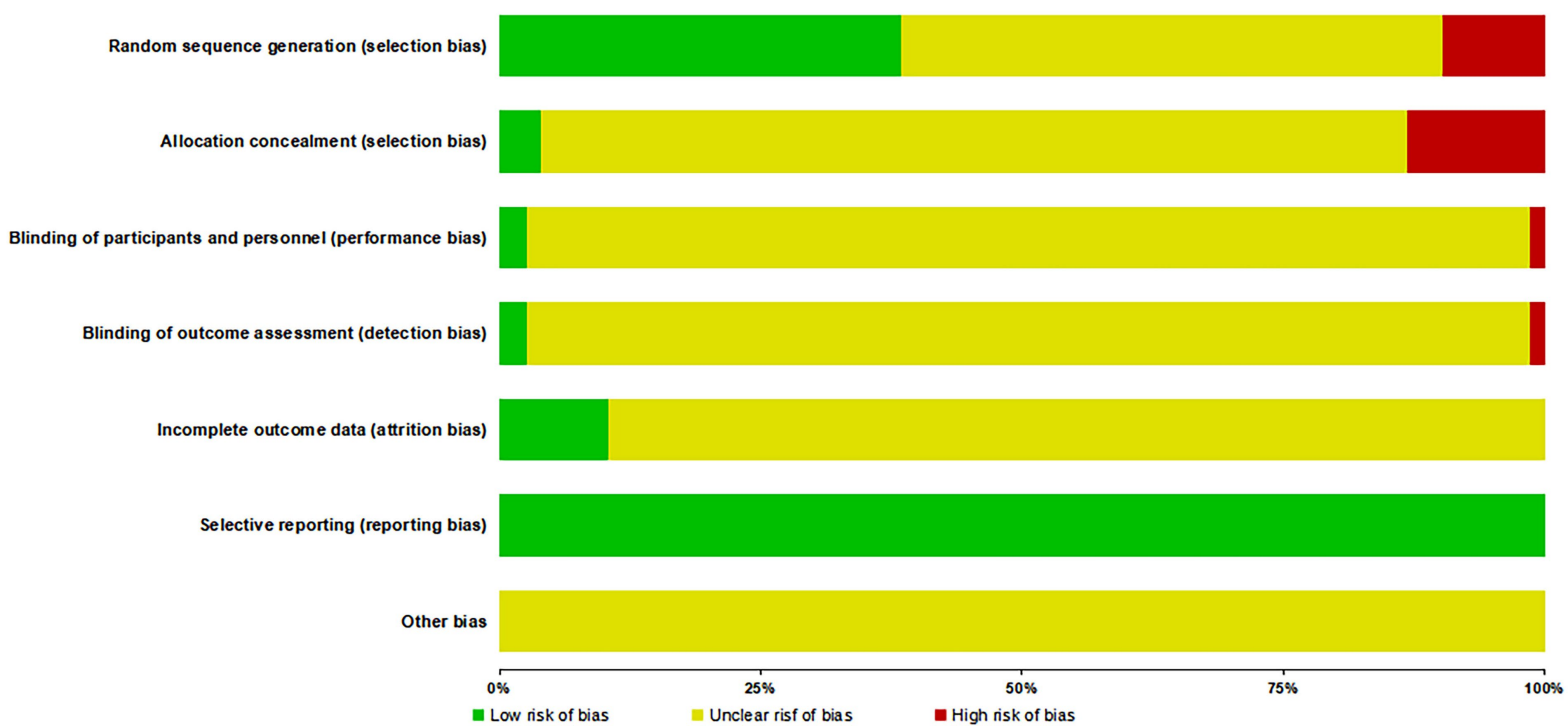
Quality assessment of RCTs on TCM for PHN.

Among 976 analyzed RCTs, 375 (38.42%) used appropriate randomization methods (e.g., random number tables or computer-generated sequences) and were rated as low risk. In contrast, 96 studies (9.84%) were deemed high risk due to flawed grouping strategies such as assignment by visit order or parity. The majority (505 studies, 51.74%) mentioned randomization but lacked sufficient detail, resulting in an unclear risk rating. Allocation concealment was poorly implemented: only 38 trials (3.89%) used effective methods (e.g., central allocation or sealed envelopes), whereas 129 (13.22%) applied predictable strategies (e.g., odd-even numbering or treatment-based assignment), and the remaining 809 (82.89%) did not report any concealment measures. Blinding was inadequately described: only 25 studies (2.56%) provided explicit blinding procedures, while 14 (1.43%) were high risk due to poor implementation, and the rest did not specify blinding methods. Although all studies reported pre-specified outcomes, only 102 (10.45%) fully addressed data completeness through the reporting of attrition and protocol deviations. No study discussed other potential sources of bias (Figure 10).

#### 3.7.2 Quality assessment of SRs/MAs

**Figure 11:**
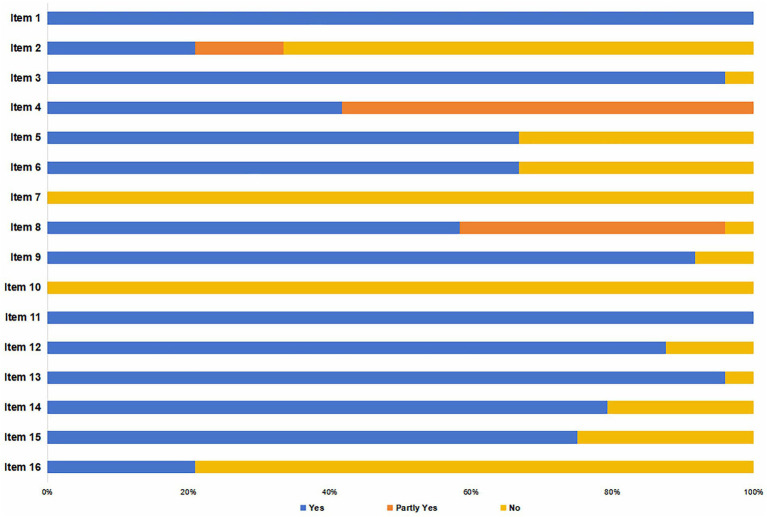
Quality assessment of SRs/MAs on TCM for PHN.

The methodological quality of the 24 included SRs/MAs was assessed using the AMSTAR-2 scale. Results indicated that 7 studies were rated as low quality and the rest as critically low. Only 12 reviews demonstrated relatively complete reporting, with ≥ 70% of items rated “yes” or “partial yes.” Satisfactory reporting was observed for Items 1, 3, 4, 8, 9, 11, 12, 13, 14, and 15. However, widespread limitations were identified: (1) critical Item 7 was severely deficient, as none provided a list of excluded studies; (2) among non-critical items, Item 10 showed notable shortcomings, with no reviews reporting the funding sources of primary studies; (3) most studies lacked a pre-registered protocol or a conflict-of-interest statement; (4) several omitted duplicate study selection or data extraction. Detailed results are presented in Figure 11 and Table 3.

**Table 3:** Summary of critical deficiencies in included SRs/MAs.

AMSTAR-2 item	Description of Deficiency	Frequency (*n*)	Percentage (%)
Item 2. Protocol established before commencement?	Failure to register/develop a protocol *a priori* (prior to starting the systematic review).	16	66.67
Item 5. Study selection by ≥ 2 independent reviewers?	Study selection (screening) not performed independently by two reviewers (nor described method of resolving disagreements).	8	33.33
Item 6. Data extraction by ≥ 2 independent reviewers?	Data extraction not performed independently by two reviewers (nor described method of resolving disagreements).	8	33.33
Item 7. Excluded literature list & reasons?	Omitted list of excluded studies at full-text stage with reasons for exclusion.	24	100.00
Item 10. Funding sources of included studies reported?	Failed to report source(s) of funding for the primary studies included.	24	100.00
Item 16. Conflicts of interest of included studies reported?	Failed to report conflicts of interest declarations for the primary studies included.	19	79.17

## 4 Discussion

This evidence-mapping study systematically evaluated RCTs and SRs/MAs on TCM interventions for PHN. Through a multidimensional analysis covering publication trends, sample sizes, TCM pattern classifications, intervention types and duration, outcome assessments, and methodological quality, it summarizes the current evidence landscape within a structured visual framework. The main findings are as follows:

### 4.1 Urgent need to improve the quality of clinical research

PHN, a common complication of HZ, is characterized by persistent and severe pain in the affected region after the herpetic lesions have healed, significantly impairing patients’ quality of life (45, 46). PHN-related pain is often intractable and refractory (45), highlighting the need for therapies with proven efficacy.

However, this evidence map reveals that the current clinical evidence supporting TCM for PHN management is generally of low quality and limited scope. More than 70% of the included RCTs were constrained by insufficient sample sizes—primarily ranging from 51 to 100 participants—which reduced their statistical power and clinical applicability for detecting treatment effects. Furthermore, critical methodological details, including randomization, allocation concealment, and blinding, were frequently inadequately reported. And all includeed SRs/MAs were rated as low (29.17%) or critically low (70.83%) quality, indicating a substantial risk of bias. Consequently, the current evidence provides limited guidance for clinical practice.

These fundamental methodological limitations undermine the reliability and generalizability of available findings. Therefore, future research should prioritize large-scale, rigorously designed multicenter RCTs with improved methodological transparency to enhance the credibility and clinical relevance of TCM interventions for PHN.

### 4.2 Unexplored potential of TCM pattern differentiation

The dynamic changes in TCM pattern are critical for assessing disease progression and treatment efficacy (35). A major advantage of TCM pattern differentiation is its capacity to accurately identify syndromes while comprehensively considering etiology, pathogenesis, lesion location, and disease nature, thereby formulating an individualized, systematic and dynamically adjusted treatment strategies to achieve precise therapeutic outcomes (47).

However, only 20% of RCTs clearly documented the TCM pattern types of the participants, and up to 95% failed to report TCM syndrome scores, which serve as a core outcome for evaluating TCM therapeutic effects. This substantial omission significantly limits the comparability and reproducibility of the findings, impeding both the evaluation and promotion of TCM pattern-specific interventions, as well as undermining the representation of TCM’s holistic and personalized approach in managing PHN.

Inconsistent criteria and definitions for syndrome diagnosis constitute a major obstacle to TCM standardization (35). Future efforts should therefore prioritize the development of well-defined, quantifiable diagnostic tools specifically for PHN-related pattern classification, followed by validation and promotion in clinical practice to improve both the reproducibility of study results. In particular, studies investigating TCM for PHN should clearly report the basis for pattern classification, the specific names of patterns identified, and the detailed diagnostic criteria applied. Such transparency is essential to facilitate cross-study comparisons and reinforce the credibility of TCM research.

### 4.3 Standardization deficiencies in outcome indicators

As the gold standard for therapeutic evaluation, RCTs require appropriate outcome indicators to ensure scientific rigor, reliability, and comparability (48). Analysis of outcome metrics from the included RCTs revealed several major issues:

1. Lack of a unified classification system: (1) Substantial heterogeneity exists in efficacy evaluation metrics: over 50% of the included trials assessed the total effective rate primarily based on reduction in pain scores, while others employed alternative endpoints such as improvement in pain symptoms (49) or TCM symptom scoring systems (50); although more than 80% of trials used the VAS to quantify pain intensity, a minority (1.42%) adopted neuropathic pain-specific instruments such as Identification pain (ID Pain), Douleur Neuropathique 4 questionnaire (DN4) and Leeds Assessment of Neuropathic Symptoms and Signs (LANSS) (51, 52). Heterogeneity in operational protocols (e.g., assessment timing, cutoff values, and data handling) directly impedes cross-study comparisons. (2) Variability exists in the number of outcome indicators: some trials focused on one or two primary endpoints (e.g., total effective rate and pain intensity), whereas others incorporated multidimensional outcomes encompassing quality of life, neurological function, and TCM syndrome scores. This inconsistency complicates comparative analysis and meta-analytical interpretation.

To date, no systematic analysis has evaluated outcome measures across PHN-related RCTs. We recommend that future research develop a core outcome set (COS) specific to PHN, established through a standardized Delphi consensus process with active patient involvement. This COS should incorporate multidimensional domains such as pain characteristics, functional recovery, biomarker profiles, and economic metrics. Widespread adoption of this COS in subsequent clinical trials will improve comparability, strengthen clinical relevance, and facilitate more robust evidence synthesis in PHN research.

2. Limitations of outcome measures in TCM-based PHN treatment: current RCTs on TCM interventions for PHN have primarily focused on total effective rate, pain intensity, and safety evaluation, while paying insufficient attention to other critical outcomes such as pain area, self-care ability, and neurotrophic factors. (1) Pain area: as an important indicator of PHN severity, pain area reflects both the spatial distribution of pain and the subjective experience of patients (53). Existing TCM studies have primarily assessed pain intensity and duration, while systematic documentation of changes in pain area remains lacking. This omission may lead to incomplete efficacy assessments. Future studies should incorporate quantitative measurements of pain area—for example, by integrating the VAS with skin sensory abnormality mapping techniques—to objectively monitor reductions in pain area among PHN patients receiving TCM treatment. (2) Self-care ability: chronic severe pain in PHN significantly impairs patients’ daily functioning and self-care ability, greatly reducing their quality of life (54). Although TCM demonstrates potential advantages in alleviating pain (55), most studies have not adequately assessed its effect on restoring self-care capacity. Future research should incorporate standardized assessment tools, such as the Barthel Index or the Functional Independence Measure (FIM), to objectively evaluate functional recovery, thereby strengthening the evidence for the holistic efficacy of TCM in managing PHN. (3) Neurotrophic factors: as secretory proteins that regulate neuronal survival, development, and function within both the central and peripheral nervous systems, neurotrophic factors play essential roles in neural regeneration and repair (56). Given that PHN represents a complex and refractory neuropathic pain condition, the modulation of these factors is of particular clinical relevance (57). Preliminary evidence suggests that TCM may contribute to neural recovery by modulating the internal microenvironment and enhancing neurotrophic factor expression (58). However, current clinical studies on TCM for PHN have largely overlooked dynamic changes in key neurotrophic factors—such as NGF and BDNF—and have seldom examined their correlation with treatment outcomes. Future research should prioritize longitudinal monitoring of neurotrophic factor levels in serum or local tissues to clarify the mechanisms underlying TCM interventions and to identify potential biomarkers for objective efficacy assessment.

### 4.4 Limitations

This study has several limitations: First, the literature search was restricted to Chinese and English databases, and over 95% of included studies were published in Chinese. This may introduce language and publication biases, potentially overestimating the benefits of TCM for PHN while omitting negative or neutral results from other languages or unpublished sources. To address these issues, the following measures are recommended: 1) prospectively register all clinical trials for PHN in international trial registries (e.g., ClinicalTrials.gov, ISRCTN, or ChiCTR); 2) include additional databases such as Japan Centra Revuo Medicina (JCRM) and KoreaMed in future reviews, and involve researchers fluent in relevant languages; 3) ensure all results are published regardless of outcome. Additionally, only focused on RCTs and SRs/MAs but excluded real-world evidence and observational studies, resulting in incomplete evidence synthesis and potentially compromising clinical generalizability. Finally, the overall methodological quality of the included studies was predominantly low, further reducing the reliability of the conclusions.

## 5 Conclusion

As the first evidence mapping on TCM interventions for PHN, this study suggests that while TCM shows potential benefits in managing PHN, significant challenges remain. Future research should prioritize rigorously designed, large-scale, multicenter clinical trials using a standardized COS to address current methodological limitations. Furthermore, improving the methodological and reporting quality of SRs/MAs is essential to promote the integration of TCM practices and evidence-based medicine for PHN treatment.

## Data Availability

The original contributions presented in the study are included in the article/Supplementary material, further inquiries can be directed to the corresponding author.
